# Moderating effects of regional disparities on the relationship between individual determinants and public health service utilization among internal migrants: evidence from the China migrant dynamic survey in 2017

**DOI:** 10.1186/s12889-022-12870-1

**Published:** 2022-03-22

**Authors:** Zhen Yang, Cheng-hua Jiang, Jiansheng Hu

**Affiliations:** 1 School of Medicine, Tongji University, No 1239 Siping Road, Yangpu District, Shanghai, 200092 China; 2 School of Medicine, Jinggangshan University, Jian, 343009 Jiangxi China

**Keywords:** Internal migrants, Regional disparity, Individual determinants, Public health services, China

## Abstract

**Background:**

Regional disparities and individual determinants have a significant impact on the accessibility of national essential public health services (NEPHS) for internal migrants (IMs) Nevertheless, few studies have explored the interaction between these two factors.

**Method:**

A cross-sectional sample of 102,632 IMs from the 2017 China Migrant Dynamic Survey was selected. The 28 provinces were divided into high-income provinces (HIPs) and low and middle-income provinces (LMIPs) according to their per capita disposable income (PCDI). Logistic regression was conducted using sex, residence duration, education, community type, migration range, social participation and relative personal income as independent variables, NEPHS awareness and health records registration (HRR) as dependent variables, and regional economic development level (REDL) as a moderating variable.

**Results:**

The rate of NEPHS awareness and HRR in HIPs (60.7, 30.6%) were lower (x^2^ = 42.486, *p* < 0.001; x^2^ = 25.573, *p* < 0.001) than those in LMIPs (62.9, 32.2%). After controlling for other variables, NEPHS awareness (OR = 1.379, *p* < 0.001) and HRR (OR = 1.661, *p* < 0.001) of IMs in HIPs were higher. Sub-group proportion of education Ms. in HIPs were higher. Sub-group = 1.379, *p* < 0.001) and HRR dependent mong internal migrants:, 61.0, 42.2%) were higher than those in LMIPs (60.4, 19.7, 35.8, 25.5%). Among urban communities, intra-provincial migration, social participation, education > 9 years, and middle-income, the protective effect of the first three factors on NEPHS awareness was greater in HIPs (OR = 1.386, *p* < 0.001; OR = 1.383, *p* < 0.001; OR = 2.008, *p* < 0.001) than in LMIPs (OR = 1.053, *p* < 0.001; OR = 1.109, *p* < 0.001; OR = 1.861, *p* < 0.001), while the effect of all five factors on HRR was greater in HIPs (OR = 1.440, *p* < 0.001; OR = 1.380, *p* < 0.001; OR = 1.895, *p* < 0.001; OR = 1.148, *p* < 0.001; OR = 1.146, *p* < 0.001) than in LMIPs (OR = 1.045, *p* < 0.05; OR = 1.169, *p* < 0.001; OR = 1.677, *p* < 0.001; OR = 1.027, *p* > 0.05; OR = 1.028, *p* > 0.05).

**Conclusions:**

REDL directly affected the NEPHS utilization of IMs, and the negative effects of vulnerable characteristics on the NEPHS utilization of IMs were amplified in HIPs. The government is urged to regard IMs with vulnerable characteristics in HIPs as the key population in future NEPHS equalization and take targeted measures to stimulate their enthusiasm to participate in NEPHS.

## Background

Social development drives global mobility. Migration can be internal or international, with the internal migrants (IMs) surpassing the number of international migrants by four times [1]. Due to institutional, logistical, linguistic and cultural barriers, IMs in some countries are not treated equally as natives, and they face many obstacles in health resources utilization in particular [2]. Studies from China, India, South Africa, and other countries show that IMs have insufficient access to local public health services [3–8]. IMs consistently under-use health services both in their original communities and in their destination cities, resulting in potential short-term and long-term health problems [9]. The effective improvement of accessibility to health services for IMs has become an important challenge globally.

During the past three decades, China has experienced the largest migration in human history, with millions of rural inhabitants moving temporarily or permanently to cities. Internal migration is inevitable and essential for the economic and social prosperity of China. IMs exceeded 240 million in 2017 [10], and the number has remained stable with a slight decline in recent years. In 2009, the central government initiated the National Essential Public Health Services (NEPHS), a service that is provided free of charge to all residents, including IMs who have lived there for more than six months [11]. NEPHS includes health records registration (HRR), health education, immunization for children and chronic disease management. Since 2009, the government has successively introduced measures to strengthen the equalization of NEPHS [12–14]. Efforts have been successful, as from 2014 to 2015, the rate of HRR increased from 22.98%t to 29.10%, and the acceptance rate of health education increased from 70.14 to 90.70% [15]. The new situation promotes the working mode of the government to change from general service to targeted service, and the focus has gradually shifted to achieving NEPHS equalization within IMs [14].

According to the model presented by Andersen and Newman [16], factors that affect health services accessibility include: (1) health services system, (2) societal determinants, and (3) individual determinants. The majority of existing studies have focused on individual determinants. Variables such as urban community, long residence duration, high education, high income, and intra-provincial migration, are considered to have a positive impact on NEPHS accessibility [3, 4, 15, 17–26]. The disadvantageous socioeconomic status (SES) in women limits their access to health services in some countries [5, 7], in contrast to China [3, 4, 15, 17, 20–26]. Previous studies have explored the impact of region disparity on the health service utilization of IMs, but the conclusions are inconsistent. Some studies have noted that the health service utilization is low in eastern China and high in central and western China [19–23, 26]. Since most of the eastern regions are high-income provinces (HIPs), while most of the central and western regions are low- and middle-income provinces (LMIPs), these studies speculated that the regional economic development level (REDL) may be negatively related to the NEPHS utilization level in IMs [15, 26]. Following comparison of the NEPHS utilization level in IMs in three developed regions (i.e., Yangtze River Delta, Pearl River Delta and Bohai Bay) with other regions, Yang [4] postulates that REDL can promote the NEPHS utilization level in IMs.

The following problems exist with the existing studies and their discussion of REDL and NEPHS utilization level. First, inferring the relationship between REDL and NEPHS utilization level indirectly through the method of dividing regional types by geographical scale is not realistic, because it ignores the REDL differences among provinces within the same geographical area. For example, in the Yangtze River Delta, per capita disposable income (PCDI) in Shanghai was nearly twice that of Anhui province in 2017, and a gap of such proportions may interfere with the analysis. Second, when analyzing regional differences using national samples, the majority of studies analyze individual determinants and REDL as independent variables, without considering the possible interactions between the two [19–21, 23, 26]. REDL should be regarded more as a societal determinant, and the effect of individual determinants on health service utilization could be influenced by societal determinants [16]. A number of transnational studies have pointed out the presence significant differences among countries in the relationship between SES and health service utilization [27, 28]. However, Pevalin [29] believes that no significant difference exists in the strength of this relationship between poor and rich areas in the UK.

According to 2017statistics, PCDI in Zhejiang province was almost 3.2 times that of Gansu province in China. Wang et al. [30] have pointed out the great differences in the mode of influencing factors of medical service utilization in these two provinces, and the income inequality in Zhejiang province is greater. Few studies have provided information about the impact of the huge regional economic imbalance on the public health service utilization of IMs in China, and whether the relationship between individual determinants and public health service utilization of IMs is different among regions with different levels of economic development. These are two crucial bottlenecks for the government to continue to promote the equalization of public health services for IMs. To provide further information and promote our understanding regarding these issues, this study divided 28 provincial-level administrative regions into HIPs and LMIPs according to PCDI, based on the 2017 China Migrant Dynamic Survey. Logistic regression analysis was conducted with the rate of NEPHS awareness and HRR as the dependent variables and individual determinants as the independent variables, to investigate the potential existence of significant regional differences in the relationship between individual determinants and NEPHS utilization level. The present study provides a new reference for promoting equalization of public health services for IMs not only in China, but also in other countries and regions with similar characteristics of unbalanced economic development.

## Methods

### Data

This is a retrospective cross-sectional study. The data was obtained from the China Migrant Dynamic Survey in 2017 provided by the Migrant Population Service Center. The China Migrant Dynamic Survey is an annual national sample survey of the IMs organized by the National Health Commission from 2009, with an annual sample size of approximately 200,000 households. The layered, multi-stage, and probability proportional to size (PPS) sampling method has been adopted in the China Migrant Dynamic Survey. This study adopted the individual questionnaire A of the China Migrant Dynamic Survey, which was uniformly printed and distributed by the National Health Commission. Questionnaire A includes basic information about demography, perception of the destination, state of social interaction, utilization status of NEPHS, etc. Full-time investigators collected the questionnaire data through household interviews, and informed consent was signed by each respondent before commencing the interview. Dates were entered through the migrant population health and household planning dynamic monitoring system, and input data were subjected to multiple checks to ensure their quality.

The target population of China Migrant Dynamic Survey is the inflow population aged 15 and above who migrated in the local area (county or city) 1 month before the survey. In this study, the sample inclusion conditions were set as “18-59 years of age, residence duration more than one year, and personal monthly income ≥0”. Beijing, Tianjin and Shanghai have only inter-provincial IMs, which do not meet the objective of this study, so the samples from these three cities were excluded. After the quality audit, 102,632 people were finally included. In addition, the provincial PCDI was introduced to reflect the REDL of each provincial region, and was based on 2017 data from the National Bureau of Statistics.

### Measurements

#### Dependent variables

Awareness of NEPHS is a prerequisite for NEPHS utilization [31], and HRR is highly effective evidence of IMs’ access to local NEPHS. Consequently, the two indicators were adopted to reflect the IMs’ accessibility to NEPHS. Awareness of NEPHS was set as an outcome variable, and the question was “Have you heard of the NEPHS?” with a binary answer of “yes or no”. Another outcome variable was HRR, which is one of the service priorities and reflects the actual utilization of NEPHS by the IMs. IMs can voluntarily register for a health record account at the local community health service center, and their health information will be recorded. The question was “Have you registered health records at the destination?” with a binary answer of “yes or no”.

#### Independent variable

Seven variables based on the literature were selected as the individual determinants, including sex, residence duration, education, community type, migration range, social participation and relative personal income. Regional disparity was measured by the REDL. Residence duration was divided into two groups, namely ≤3 years and > 3 years. According to years of compulsory education in China, education was divided into two groups, namely ≤9 and > 9 years groups. Community types were divided into urban and rural communities. The migration range was divided into inter-provincial and intra-provincial migration.

Different criteria for regional disparity classification from previous studies were adopted in this study. PCDI was introduced to measure REDL, and REDL was divided into two groups according to PCDI in 2017. Five provinces (Zhejiang, Jiangsu, Guangdong, Fujian, and Shandong) with PCDI of more than RMB 36000 were classified as HIPs, and the remaining 23 provincial districts were classified as LMIPs.

Personal income is a widely discussed factor affecting the accessibility of health services. Nevertheless, most existing studies have used absolute income [5, 7, 15, 18–20, 22, 23]. The unbalanced economic development of various provinces in China means that a person earning 3000 Yuan per month is considered to belong in the high-income group in Gansu province, but a low-income group in Zhejiang. Wilkinson [32] pointed out that relative income is a better indicator of SES, and Chen & Carol [33] mentioned that relative income is a more effective variable when analyzing regional health inequality in the United States. Therefore, the concept of relative income was adopted in this study, with relative personal income being equal to personal income divided by provincial PCDI. Relative personal income was further divided into three categories: low (0–0.99), middle (1.00–1.99) and high (≥2).

Social participation is an important means to obtain health information [18, 24, 34], and was also included as one of the independent variables in this study. The question of social participation was “Have you participated in any of the following activities in the past year: trade unions, volunteer associations, homecoming associations, fellow-students association, home town chamber of commerce, others?”, with a binary answer of “yes or no”.

### Statistical analysis

First, the distribution characteristics of all included variables were described. Then, cross table and Chi-square test were employed to detect significant differences in the population structure of IMs between HIPs and LMIPs (Table 1). Then, for the overall sample, cross table and Chi-square test were used again to verify whether the included independent variables had a significant impact on the dependent variables. The total sample was divided into two groups (HIPs and LMIPs), and cross-table analysis and Chi-square test were conducted on the two sub-samples, respectively, to verify whether individual determinants had significant impact on the dependent variables in each group (Table 2), and whether there was any difference in the impact of the individual determinants on dependent variables between the two groups. Hierarchical logistic regression analysis was performed, following univariate analysis (Table 3). The dependent variables of the logistic regression model were awareness of NEPHS and HRR. The independent variables of the first-level model included sex, residence duration, education, community type, migration range, social participation, relative personal income and REDL. The interaction terms of REDL and other seven independent variables were added to the second-level model. Finally, a grouping logistic regression analysis was conducted using awareness of NEPHS and HRR as independent variables, sex, residence duration, education, community type, migration range, social participation and relative personal income as independent variables, and REDL as a grouping variable (Table 4). Sampling weights were included in all analyses to adjust for the complexity of the survey design. In logistic regression analysis model, odds ratios (OR) are presented (Tables 3 and 4). All the analyses were performed using SPSS 22.0.

**Table 1:** The composition difference of sub-groups of sex, residence duration, education, community type, migration range, social participation, and relative personal income between two groups of region economic developing leve (*N* = 102,632)

Variables	Subgroups	Total		HIPs		LMIPs		*Χ*^*2*^*, p*
		N	%	N	%	N	%	
Awareness of NEPHS	No	38,742	37.7	11,472	39.3	27,270	37.1	42.486, *p* = 0.000
	Yes	63,890	62.3	17,709	60.7	46,181	62.9	
Health records registration	No	70,042	68.2	20,255	69.4	49,787	67.8	25.573, *p* = 0.000
	Yes	32,590	31.8	8926	30.6	23,664	32.2	
Sex	Male	58,647	57.1	16,426	56.3	42,221	57.5	12.111, *p* = 0.001
	Female	43,985	42.9	12,755	43.7	31,230	42.5	
Residence duration	≤3 years	30,077	29.3	8966	30.7	21,111	28.7	39.674, *p* = 0.000
	> 3 years	72,555	70.7	20,215	69.3	52,340	71.3	
Education	≤9 years	62,632	61.0	18,271	62.6	44,361	60.4	43.169, *p* = 0.000
	> 9 years	40,000	39.0	10,910	37.4	29,090	39.6	
Community type	Urban	77,466	75.5	18,488	63.4	58,978	80.3	3237.816, *p* = 0.000
	Rural	25,166	24.5	10,693	36.6	14,473	19.7	
Migration range	intra-province	58,526	57.0	11,375	39.0	47,151	64.2	5417.292, *p* = 0.000
	inter-province	44,106	43.0	17,806	61.0	26,300	35.8	
Social participation	No	53,636	52.3	14,782	50.7	38,854	52.9	42.061, *p* = 0.000
	Yes	48,996	47.7	14,399	49.3	34,597	47.1	
Relative personal income	Low	30,891	30.2	12,281	42.2	18,610	25.5	3900.582, *p* = 0.000
	Middle	47,325	46.3	13,077	44.9	34,248	46.9	
	High	23,963	23.5	3736	12.9	20,229	27.7	

Note:(1) ***p < 0.001, **p < 0.01, *p < 0.05. (2) *HIPs *high income provinces, *LMIPs *low-and middle- income provinces; (3) *NEPHS *National Essential Public Health Services

**Table 2:** The interactions between REDL and sex, residence time, education, community type, migration range, social participation and relative personal income on utilization of NEPHS(*N* = 102,632)

Variables	Subgroups	Rate of awareness of NEPHS (%)				Rate of health records registration (%)			
		Total	HIPs	LMIPs	*Χ*^*2*^*, p*	Total	HIPs	LMIPs	*Χ*^*2*^*, p*
Sex	Male	61.2	60.3	61.6	0.941, *p* = 0.332	30.9	29.4	31.5	12.650, *p* = 0.000
	Female	63.6	61.2	64.6		32.8	32.1	33.1	
Residence duration	≤3 years	62.4	59.1	63.9	3.168, *p* = 0.075	30.7	29.9	31.0	18.056, *p* = 0.000
	> 3 years	62.2	61.4	62.5		32.2	30.9	32.7	
Education	≤9 years	58.6	57.3	59.1	28.939, *p* = 0.000	30.0	27.5	31.0	9.268, *p* = 0.002
	> 9 years	67.9	66.3	68.6		34.6	35.8	34.1	
Community type	Urban	63.9	65.0	63.6	1302.940, *p* = 0.000	33.1	34.6	32.6	352.507, *p* = 0.000
	Rural	57.2	53.2	60.1		27.7	23.7	30.7	
Migration range	intra-province	64.8	67.3	64.2	2644.562, *p* = 0.000	34.2	36.6	33.6	1129.471, *p* = 0.000
	inter-province	58.9	57.5	60.5		28.5	26.7	29.7	
Social participation	No	54.5	51.9	55.6	61.607, *p* = 0.000	25.8	23.3	26.8	78.782, *p* = 0.000
	Yes	70.7	69.7	71.1		38.2	38.1	38.3	
Relative personal income	Low	59.4	57.9	60.4	2213.865, *p* = 0.000	30.1	27.9	31.6	911.543, *p* = 0.000
	Middle	63.4	62.3	63.8		32.6	32.3	32.7	
	High	63.7	64.2	63.6		32.2	33.3	32.0	

Note: (1) ***p < 0.001, **p < 0.01, *p < 0.05. (2) *HIPs *high income provinces, *LMIPs *low-and middle- income provinces, *NEPHS*: National Essential Public Health Services

**Table 3:** Logistic regression results of sex, residence duration,education, community type, migration range, social participation, region economic developing leve and relative personal income on utilization of NEPHS (*N* = 102,632)

Independent Variables	Reference group	Awareness of NEPHS *(OR)*		Health records registration *(OR)*	
		*Block 1*	*Block 2*	*Block 1*	*Block 2*
Sex	Female	1.159***	1.104***	1.124***	1.221***
	Male				
Residence duration	> 3 years	1.023	1.099***	1.096***	1.048
	≤3 years				
Education	> 9 years	1.262***	1.140***	1.060***	1.148***
	≤9 years				
Community type	Urban	1.173***	1.386***	1.187***	1.440***
	Rural				
Migration range	Intra-province	1.193***	1.383***	1.246***	1.380***
	Inter- province				
Social participation	Yes	1.901***	2.008***	1.729***	1.895***
	No				
Relative personal income	Low	1.096***	1.081**	1.069***	1.146***
	Middle				
	High	1.078***	1.032	1.034	1.076
REDL	LMIPs	1.020	1.397**	1.000	1.661***
	HIPs				
REDL*Sex			1.068*		0.896**
REDL*Residence duration			0.897**		1.056
REDL*Education			1.144***		0.895**
REDL*Community type			0.759***		0.726***
REDL*Migration range			0.802***		0.847***
REDL*Social participation			0.927*		0.885***
REDL*Relative personal income (middle)			1.009		0.897**
REDL*Relative personal income (high)			1.042		0.924

Note: (1) ***p < 0.001, **p < 0.01, *p < 0.05. (2) *HIPs *high income provinces, *LMIPs *low-and middle- income provinces, *NEPHS *National Essential Public Health Services, *REDL *region economic developing leve

**Table 4:** Logistic regression results of sex, residence duration,education, community type, migration range, social participation, and RPCDI on utilization of NEPHS in two groups of region economic developing level (*N* = 102,632)

Independent Variables	Reference group	Awareness of NEPHS [*OR,* 95%CI]		Health records registration [*OR,* 95%CI]	
		Model 1 (HIPs)	Model 2(LMIPs)	Model 3(HIPs)	Model 4(LMIPs)
Sex	Female	1.104*** [1.049, 1.162]	1.179*** [1.142,1.218]	1.221*** [1.157, 1.289]	1.094*** [1.059, 1.131]
	Male				
Residence duration	> 3 years	1.099*** [1.043, 1.158]	0.985 [0.952,1.020]	1.048 [0.991, 1.108]	1.106*** [1.068, 1.146]
	≤3 years				
Education	> 9 years	1.140*** [1.081,1.203]	1.305*** [1.263,1.348]	1.148*** [1.086,1.213]	1.027 [0.994,1.062]
	≤9 years				
Community type	Urban	1.386*** [1.316, 1.460]	1.053** [1.013, 1.094]	1.440*** [1.360, 1.525]	1.045* [1.004, 1.088]
	Rural				
Migration range	Intra-province	1.383*** [1.313,1.456]	1.109*** [1.074, 1.145]	1.380*** [1.309, 1.455]	1.169*** [1.130, 1.208]
	Inter-province				
Social participation	Yes	2.008*** [1.911,2.109]	1.861*** [1.804,1.921]	1.895*** [1.799, 1.997]	1.677*** [1.624,1.732]
	No				
Relative personal income	Low	1.084** [1.027, 1.144]	1.093*** [1.052,1.135]	1.146*** [1.082, 1.214]	1.028 [0.989,1.070]
	Middle				
	High	1.032 [0.950, 1.120]	1.074** [1.029,1.122]	1.076 [0.988, 1.172]	0.994 [0.950,1.040]

Note: (1)***p < 0.001, **p < 0.01, *p < 0.05.(2) *HIPs *high income provinces, *LMIPs *low-and middle- income provinces, *NEPHS *National Essential Public Health Services

## Results

### Differences of the IMs’ population characteristics between HIPs and LMIPs

Table 1 shows that the awareness of NEPHS was 62.3%, while the HRR rate was 31.8%. The awareness of NEPHS and HRR rates of the IMs in LMIPs were significantly higher than those in HIPs. In terms of total sample composition, the proportion of IMs was higher among males, resident duration > 3 years, education ≤9 years, urban communities, inter-provincial migration and those from LMIPs. The social participation of the IMs was low, with 52.3% of them not participating in any of the organizations listed in the questionnaire in the past year. The relative personal income of the IMs was relatively low. The IMs whose personal income was lower than the local PCDI accounted for 30.2%, and the middle and low income groups accounted for 76.5% of the total sample.

Table 1 also shows differences of the IMs’ characteristics in sex, residence duration, education, community type, migration range, social participation and relative personal income between HIPs and LMIPs groups. The population structure of IMs in HIPs was significantly different from the corresponding in LMIPs. Compared with LMIPs, there was a higher proportion of sub-group of females (43.7% VS 42.5%), resident duration ≤3 years (30.7% VS 28.7%), education ≤9 years (62.6% VS 60.4%), rural community (36.6% VS 19.7%), inter-provincial migration (61.0% VS 35.8%), social participation (49.3% VS 47.1%) and low-income (42.2% VS 25.5%) in HIPs.

### Univariate analysis of the impacts of individual determinants on NEPHS utilization

Table 2 shows that female IMs (Χ^2^ = 59.269, *p* = 0.000), residence duration > 3 years (Χ^2^ = 0.711, *p* = 0.399), education > 9 years (Χ^2^ = 903.376, *p* = 0.000), urban community (Χ^2^ = 367.754, *p* = 0.000), intra-province migration range (Χ^2^ = 372.441, *p* = 0.000), having social participation (Χ^2^ = 2837.832, *p* = 0.000), high relative personal income (*Χ*^2^ = 149.369, *p* = 0.000) have a higher rate of NEPHS awareness; female IMs (*Χ*^2^ = 41.581, *p* = 0.000), residence duration > 3 years (*Χ*^2^ = 22.883, *p* = 0.000), education > 9 years (*Χ*^2^ = 240.661, *p* = 0.000), urban community (*Χ*^2^ = 251.392, *p* = 0.000), intra-province migration range (*Χ*^2^ = 381.242, *p* = 0.000), having social participation (*Χ*^2^ = 1816.324, *p* = 0.000), middle relative personal income (*Χ*^2^ = 56.585, *p* = 0.000) have a higher rate of HRR. Table 2 also reveals that there were significant differences in NEPHS awareness rates between the HIPs and LMIPs group in different sub-groups of five independent variables, in addition to sex and residence duration: education, community type, migration range, social participation and relative personal income. However, the HRR rates of all independent variable subgroups were significantly different between the HIPs and LMIPs groups. This difference was highlighted in three independent variables: community type, migration range and relative personal income.

### Analysis of the moderating effect of REDL

When awareness of NEPHS was taken as the dependent variable, Omnibus test Χ^2^ increased from 3753.259 (*p* < 0.001) to 3917.690 (*p* < 0.001), Cox & Snell R^2^ increased from 0.036 to 0.038, Hosmer & Lemeshow test Χ^2^ decreased from 10.911 (*p* > 0.05) to 6.825 (p > 0.05) for block 2 versus block 1, respectively (Table 3). With HRR as the dependent variable, Omnibus test Χ^2^ increased from 2382.530 (*p* < 0.001) to 2599.108 (*p* < 0.001), Cox & Snell R^2^ increased from 0.023 to 0.025, Hosmer & Lemeshow test Χ^2^ decreased from 36.594 (*p* < 0.001) to 6.169 (*p* > 0.05) for block 2 versus block 1, respectively. These results show that the interaction effect model has better statistical power. Grouping logistic regression analysis results according to REDL shown in Table 4 showed significant differences between the HIPs (Model 1 and 3) and LMIPs (Model 2 and 4) groups.

Table 3 shows that the main effects of seven variables (i.e., sex, residence duration, education, community type, migration range, social participation and relative personal income) on NEPHS awareness were significant (OR values were greater than 1), while the main effects of seven variables (i.e., sex, education, community type, migration range, social participation, relative personal income and REDL) on HRR were significant (OR values were greater than 1). Interaction effects between REDL and six other variables (i.e., sex, residence duration, education, community type, migration range and social participation) on awareness of NEPHS were significant, and interaction effects between REDL and six variables (i.e., sex, education, community type, migration range, social participation and middle income) on HRR were significant (OR values were less than 1). Table 4 shows that OR values of females, residence duration > 3 years of education > 9 years, urban community, intra-provincial migration, social participation, and middle income were greater than 1 in Model 1, and in Model 2, some OR values of corresponding terms were larger and some were smaller. The OR values of females, education > 9 years, urban community, intra-provincial migration, social participation, and middle income were greater than 1 in Model 3, and the OR values of the corresponding terms in Model 4 were all smaller.

## Discussion

Twenty-eight provincial regions in China were divided into HIPs and LMIPs according to PCDI in this study. This new classification produced different results from previous studies. Compared with LMIPs, the NEPHS utilization level of IMs in HIPs was lower and the gap more prominent after controlling for other variables and interaction effects, while the proportion of IMs with vulnerable characteristics was higher and the negative effects of these characteristics on service access were stronger.

Previous studies have attributed the low level of IMs’ NEPHS utilization in HIPs to the shortage of NEPHS resources per capita [15, 22, 26] Although the samples from Beijing, Tianjin and Shanghai were excluded, our results partly support this conclusion [19–23, 26], that is, IMs in HIPs have lower levels of NEPHS utilization. The relationship between REDL and health service utilization seems to be at odds with what is commonly believed. The HIPs have higher NEPHS resources than LMIPs [35, 36], but the HIPs have absorbed more than 70% of the inter-provincial IMs [10], thus the influx of inter-provincial IMs has diluted its per capita public health resources. Meanwhile, public health resources in LMIPs have grown rapidly with the help of transfer payments from the central government. As a result, public health resources per capita in HIPs are relatively inadequate. Data from 2017 showed that the developed Yangtze River Delta had 4.01 primary medical institutions per 10,000 people, while the average in other parts of China was 7.52 [37]. In this study, after controlling for some variables, especially the interaction between REDL and other variables, the disadvantage of NEPHS accessibility of IMs in HIPs was further amplified. The results of our study seem to further confirm that the lack of per capita resources is an important factor restricting the access of IMs to NEPHS in HIPs.

The difference in the IMs’ population structure may be another important reason for the gap in access to NEPHS between HIPs and LMIPs. Factors involving supply play important roles in individual health care utilization, but do not fully explain geographical inequalities [38]. Just as Andersen and Newman [16] pointed out that both the volume and distribution of resources have a high impact on the accessibility of health services, distribution of NEPHS resources among the IMs in HIPs has been difficult. The results revealed that the proportion of subgroups with residence duration ≤3 years, education ≤9 years, rural community, inter-provincial migration and low income was significantly higher in HIPs. In fact, these characteristics were risk factors for NEPHS utilization, both in previous studies [3, 4, 15, 17–26] and in the present study. Low education and income mean low SES, and low SES is a risk factor for health service utilization [39]. Short-term residence and inter-provincial migration mean that IMs will face more cultural conflicts and adaptation difficulties, which further affects the social network construction of the IMs in the destination, and the social network is an important channel for migrants to access health service information [40]. The urban-rural dual economic structure in China makes public health resources in rural communities poorer than those in urban communities, which can result in a reduction of IMs’ NEPHS utilization level in HIPs for a high proportion of vulnerable groups.

In HIPs, the proportion of IMs with vulnerable characteristics was higher, in addition to the negative effect of these characteristics on IMs’ NEPHS utilization being stronger. Among migration range, community type, social participation, education and income, the effects of the first three variables on NEPHS awareness were stronger in HIPs, while effects of all five variables on HRR were stronger in HIPs. Compared with LMIPs, the subgroup of IMs with intra-provincial migration, high-income and urban community had a higher NEPHS utilization level, while the subgroup of IMs with inter-provincial migration, low-income and rural community had a lower NEPHS utilization level in the HIPs. These results show that access to NEPHS was less equally distributed among IMs in HIPs than in LMIPs. Although this finding is different from the report of Pevalin [29], it is basically consistent with the results of Wang et al. [30]. This may be due to the different hukou registration systems adopted by HIPs and LMIPs. The HIPs implement a relatively high entry threshold in order to control the population size, while the LMIPs adopt more inclusive policies to attract IMs. It is more difficult for disadvantaged IMs to obtain hukou in HIPs, and many rights are still tied to hukou. The main obstacles for the IMs to obtaining citizenship, equal social rights and political participation are institutional factors [41]. Institutional exclusion could weaken the IMs’ identification and belonging to the destination, and which could further reduce their enthusiasm to participate in NEPHS, resulting in enhancement of the negative effects of vulnerable characteristics. Just as Yang Xin [4] said, only paying attention to supply factors without stimulating IMs’ participation cannot promote the NEPHS equalization to a higher level.

The results of this study prompted the Chinese government to focus on the disadvantaged groups in HIPs in the future work of equalization of NEPHS for IMs. In view of the high proportion of vulnerable IMs, it is necessary for HIPs to adopt different strategies to promote the equality of NEPHS from LMIPs. In this regard, we proposed three suggestions: (1) First, HIPs need to remove institutional barriers for IMs to access NEPHS resources, and gradually remove social welfare and resource allocation functions attached to the household registration system. (2) HIPs should take measures to push the input of public health service resources for rural communities, and reduce the gap of NEPHS between urban and rural areas as soon as possible. (3) HIPs need to perform well in community mobilization, strengthen the registration system of IMs, and timely grasp the IMs’ information for classified management. Targeted help, publicity and mobilization should be organized and performed for the disadvantaged migrant population, to stimulate the enthusiasm of the vulnerable groups to participate in NEPHS.

### Strength and limitations

Different from previous studies, PCDI instead of geographical scale was adopted in this study to classify 28 provincial regions into HIPs and LMIPs, and excluded samples from Beijing, Shanghai and Tianjin to reduce bias. The results from this innovative way of classification yielded three highlights: (1) The re-examining of the gap of IMs’ NEPHS utilization between HIPs and LMIPs deepened our understanding. (2) The results point that the population structure difference of IMs between HIPs and LMIPs may be an important reason for the NEPHS inequality of IMs in HIPs. (3) This is the first time to the knowledge of the authors that the negative effects of vulnerable characteristics on the IMs’ NEPHS utilization were shown to be amplified in HIPs. At the same time, this study also had two limitations: (1) Only two indicators (NEPHS awareness and HRR) were chosen to reflect the IMs’ situation of NEPHS utilization. More programs should be discussed in detail, to increase the overall understanding of the impact of REDL on NEPHS utilization. (2) Due to limited authority, only the data of the 2017 China Migrant Dynamic Survey were analyzed, which is now more than 4 years old. In view of the rapid progress in the equalization of NEPHS for IMs in China, the data needs to be updated to draw conclusions more consistent with the current situation.

## Conclusions

This study adopted per capita disposable income (PCDI) and a new classification, to divide 28 provincial regions in China into high income provinces (HIPs) and low and middle income provinces (LMIPs). Although the results of univariate analysis showed that the internal migrants (IMs’) national essential public health services (NEPHS) utilization level in HIPs was significantly lower than that in LMIPs, and the gap was more prominent after controlling for other variables and interaction effects. This study revealed that the sub-group proportion of IMs with vulnerable characteristics was higher in HIPs than in LMIPs. Compared with LMIPs, NEPHS utilization level of IMs with vulnerable characteristics was lower, and the negative effects of these characteristics on the IMs’ NEPHS utilization were amplified in HIPs. The government is urged to regard the IMs with vulnerable characteristics in HIPs as the key population in the future NEPHS equalization policies and take targeted measures to stimulate their enthusiasm to participate in NEPHS.

## Data Availability

You can log on to the China Migrant Population Data Platform (https://www.chinaldrk.org.cn/wjw /#/home), and follow the prompts on the website to register an account for free. If you want to obtain research data, you need to submit a research plan and an application form for using data from the National Internal Migrant Dynamic Monitoring Survey to the platform. The application form needs to be authorized by the researchers’ institution. At the same time, researchers need to sign a data use agreement with the Migrant Population Service Center of the National Health Commission, guaranteeing that they will use the data in accordance with the requirements of the agreement and will not transfer the data to any third party. If researchers have research results that are published publicly, they need to upload the results to the platform account they signed up for, so that they are eligible for the next application.

## Abbreviations

HRR: Registration of Health Records
HIPs: High Income Provinces
IMs: Internal Migrants
LMIPs: Low- and Middle- Income Provinces
NEPHS: National Essential Public Health Services
PCDI: Per Capita Disposable Income
REDL: Regional Economic Development Level
SES: Socioeconomic Status
